# Sitting time and health outcomes among Mexican origin adults: obesity as a mediator

**DOI:** 10.1186/1471-2458-12-896

**Published:** 2012-10-23

**Authors:** Hendrik D de Heer, Anna V Wilkinson, Larkin L Strong, Melissa L Bondy, Laura M Koehly

**Affiliations:** 1 Department of Physical Therapy and Athletic Training, Northern Arizona University, 208 E Pine Knoll Dr. Bldg. 66, Rm. 112, PO Box 15105, Flagstaff 86011, AZ, USA; 2 Michael & Susan Dell Center for Healthy Living, The University of Texas School of Public Health Austin Regional Campus, Austin, TX, USA; 3 Department of Health Disparities, The University of Texas MD Anderson Cancer Center, Houston, TX, USA; 4 Dan L. Duncan Cancer Center, Baylor College of Medicine, Houston, TX, USA; 5 National Human Genome Research Institute, Social and Behavioral Research Branch, Bethesda, MD, USA

**Keywords:** Sitting time, Diabetes, Obesity, Hypertension, Hispanic

## Abstract

**Background:**

Sitting time and sedentary behaviors have been associated with adverse health outcomes including obesity, diabetes and cardiovascular disease (CVD) within non- Hispanic White populations. Similar associations have not been described within Hispanic populations despite their high CVD risk profile. This study aimed to assess the association between sitting time and obesity, self-reported diagnosed diabetes, hypertension and high cholesterol among a large cohort (N=11,268) of Mexican origin adults and to assess whether obesity mediated these associations.

**Methods:**

Using a cross-sectional design, data collected between 2004 and 2010 were analyzed in late 2010. Regression analyses evaluated associations between self-reported daily sitting hours and disease outcomes, controlling for demographics, employment status, family disease history, and light, moderate and strenuous physical activity.

**Results:**

Participants were mostly female (81.1%) Mexican origin adults. Sitting time was associated with increased odds of being obese, having diabetes and having hypertension, but not high cholesterol. Adjusted odds ratios of participants who reported sitting > 4 hours/day compared to those sitting 1-2 hours/day were for obesity OR=1.55 (95% CI 1.39, 1.73), *p*<.001, for diabetes OR=1.29 (95% CI, 1.09, 1.52), *p*=.003, for hypertension OR=1.17 (95% CI, 1.01, 1.37), *p*=.041. Associations controlled for physical activity and employment status. Effects on hypertension and diabetes were mediated by obesity.

**Conclusions:**

Sitting time was significantly associated with detrimental health outcomes, independent of physical activity. Obesity mediated these relationships for diabetes and hypertension. Future research should assess whether interventions addressing sitting time are feasible and effective among Mexican origin populations.

## Background

Most adults in the U.S. do not meet daily recommended amounts of physical activity and engage in large amounts of sedentary behaviors, such as commuting by automobile, sitting at work and watching TV at home [1]. Several studies have documented the association between sedentary behaviors including time spent sitting and obesity, and between sitting time and cardiovascular risk factors including hypertension, high cholesterol and diabetes [2-26]. While the associations between sitting time and health outcomes have been moderately well described in non-Hispanic White populations, to date, there is very limited research describing these associations within minority populations. Hispanics represent the largest and fastest growing U.S. minority population [27]. Investigating these associations among Hispanic populations is particularly important given their higher mean Body Mass Index (BMI) relative to national averages [28] and increased risk of developing diabetes [29] despite spending less time in sedentary activities than non-Hispanic Whites [1].

Recently, sitting time has been shown to be associated with chronic disease risk factors *independently* of engagement in physical activity and a number of other covariates including diet and smoking status [12,21]. Thus, time spent sitting may be associated with adverse health outcomes, even when recommendations for physical activity are being met [14,15]. In other words, “*too much sitting is distinct from too little exercise*” [14].

Hamilton and colleagues [21] further reported that associations of sedentary behaviors and cardiovascular or metabolic outcomes are independent of indicators of excess adiposity, suggesting that health consequences of too much sitting are not simply a result of changes in body composition. A number of studies evaluating these associations controlled for an indicator of obesity (BMI or waist circumference) [2-6,8-12]. Several of these studies found evidence that obesity status attenuated the association between sitting time and health outcomes, [2,3,8,16] although some reported the effects were small. Gaining more insight into whether obesity is an intermediate variable in the pathway between sedentary behaviors and detrimental health outcomes will be important for primary prevention efforts.

In this context, the current study aimed to evaluate the association between sitting time and obesity, and between sitting time and chronic disease outcomes including diabetes, hypertension and high cholesterol among a large cohort (N=11,268) of the largest subgroup of Hispanics: Mexican Americans. It was hypothesized that increased sitting hours per day would be associated with increased odds of being obese and having diabetes, hypertension and high cholesterol, independent of engagement in physical activity and employment status. The second aim was to assess whether obesity mediates the association between sitting time and diabetes, hypertension and high cholesterol.

## Methods

### Participants & recruitment

The Mano a Mano Cohort Study is an ongoing study of over 20,000 Mexican American participants iniated by The University of Texas MD Anderson Cancer Center (UTMDACC) Department of Epidemiology in 2001. The overarching aim of the study is to understand cancer related risk factors as they emerge in this population, and to use this information to facilitate development of cancer prevention strategies. Participants have been recruited through random- digit-dialing, block walking in predominantly Mexican American neighborhoods, from community centers and local health clinics, and networking through currently enrolled participants [30]. Once a household member agrees to participate, a pair of bilingual interviewers visits the home to consent (in English or Spanish), enroll and interview up to three adults.

Procedures are in place to facilitate recruitment and continued contact with the household, which have shown to be successful. Examples include a pre-determined structure of multiple phone calls at different times of the day, which in the case of no response are followed by mailed letters, which are followed by home visits. Due to these efforts, recruitment rates are 150 households a month [31]. The original survey, initiated in 2001, did not include items regarding employment status; these questions were added in September 2004. Due to prior research indicating the importance of employment status on health outcomes [32], we included only those participants who were asked the employment items. As a result, the current analyses are restricted to participants recruited between September 2004 and June 2010, which included cross-sectional data on a total of 11,268 non-pregnant Mexican American adult (age ≥ 18) cohort participants. Only one participant per household was included. Data were analyzed in the fall of 2010 and in the summer of 2012. All procedures were approved by the Institutional Review Board of The University of Texas MD Anderson Cancer Center.

### Measures

All data were collected during an in-person interview by a bilingual interviewer, and were self- reported.

#### Demographic characteristics

Demographic characteristics included age, sex, proportion of life lived in the U.S., educational attainment and employment status. Educational attainment was assessed by asking: “*what is the highest level of formal education that you have?*” and dichotomized into (0) *technical/vocational training or less* and (1) *at least some college*. Employment status was dichotomized into (0) *is not currently employed or has never been employed* and (1) is *currently employed*. As a proxy for acculturation, a variable was included describing the proportion of life a participant spent in the United States. This variable was calculated by dividing the answer to the question: “*How many years have you lived in the United States?*” by the participants’ age.

Hours of sleep was assessed by asking *“On average, during the last year, how many hours in a day did you usually sleep, including naps?”* with response options (1) 5 hours or less, (2) 6 hours, (3) 7 hours, (4) 8 hours, (5) 9 hours or (6) 10+ hours. Values were recoded to reflect the hours of sleeping (i.e. 6 hours of sleep for option 2).

#### Family disease history

Family history of diabetes, hypertension and high cholesterol in first-degree relatives was defined by asking: “*Please indicate the diseases* [*your parent, sibling or child*] *has or has had that were diagnosed by a doctor and for which they are/were taking medications?”* Family disease history was included in the analyses as a dichotomized predictor comparing (0) “no” or (1) “yes” for at least one first-degree blood relative with a disease history of the respective condition.

#### Physical activity

Physical activity was assessed with items adapted from the California Teachers Study, a prospective cohort of female public school teachers and administrators [33]. Participants indicated the average number of hours per week they engaged in light, moderate, and strenuous physical activities. Participants were shown a card with example activities for each category. The activity intensity categorization was based on the Metabolic Equivalent (METs) associated with light activity (<3.0 METs, such as standing, walking at a slow pace or cooking), moderate activity (3-6 METs, such as brisk walking, lifting 10-20 lb. items, mowing or raking the lawn, cycling on a flat surface) and strenuous activity (>6 METs, such as swimming laps, running or cycling on hills) [34]. Response scales for all three levels of physical activity included (1) Never; (2) 0.5 hours; (3) 1 hour; (4) 1.5 hours; (5) 2 hours; (6) 3 hours; (7) 4-6 hours; (8) 7-10 hours; (9) 11+ hours. Values were recoded to reflect the amount of hours the participants engaged in the behavior, using the median value of each interval (e.g. 5 hours for the option 4-6 hours). The hours per week were divided by seven to reflect hours per day for each variable and to make units comparable.

#### Sitting time

Daily sitting hours were assessed with items adapted from the California Teachers study by asking the participant to “*Please estimate the average number of hours [you are] sitting each day either at work, school or home.”* The item options used for the current study were (1) 1-2 hours, (2) 2-4 hours, (3) 4-6 hours, (4) 6-8 hours, (5) 8-10 hours, (6) 10-12 hours, (7) 12-14 hours, (8) 14-18 hours or (9) 18+ hours. Similar to procedures used in prior research that divided sitting time hours into categories such as low, moderate and high sitting [8] or quartiles [24], participants were categorized into three categories: those participants who reported sitting 1-2 hours/day (27.0%), sitting 2-4 hours/day (41.1%), and sitting more than 4 hours per day (32.0%).

#### Outcome variables

The outcomes consisted of obesity, diabetes status, high cholesterol and hypertension. Obesity was dichotomized based on participant BMI with obesity defined as having a BMI ≥ 30 kg/m2. BMI was calculated from self-reported height and weight. More than half of the participants (n=6,206) also had their height and weight measured in addition to self-report. The correlation between reported and measured BMI among participants was high (*r*=0.93). Disease status of the participants was assessed by asking: “*Have you ever been told by a health professional that you had diabetes/ high blood pressure/ high cholesterol*” Participants reported only those conditions diagnosed by a doctor for which they currently take, or previously took medications.

### Analyses

T-tests and Analysis of Variance (for continuous variables) and chi-square tests (for categorical variables) were used to assess differences across sex and across time spent sitting. The sitting time variable was split into three categories: (1) 1-2 hours of sitting, (2) 2-4 hours of sitting and (3) more than 4 hours of sitting. Logistic regression analyses were conducted to assess whether sitting time (as a categorical variable) was associated with increased odds of obesity, diabetes, hypertension and high cholesterol, controlling for strenuous, moderate and light physical activity and other covariates. Covariates in the logistic regression models included age, sex, proportion of life spent in the U.S., educational attainment, employment status, hours of sleep and family history of diabetes, hypertension and high cholesterol.

Approaches for mediational analyses [35] with dichotomous outcomes were used [36] to test whether obesity status mediated the association between sitting time and disease outcomes. Mediation was assumed if (a) sitting time significantly predicted obesity status, (b) sitting time significantly predicted disease outcomes without obesity status in the model, (c) obesity status predicted disease outcomes, and (d) the effect of sitting time on disease outcomes was significantly reduced when obesity status was added to the model [37]. The Sobel test was used to test this mediation through assessing whether a critical ratio from the independent variable on the dependent variable through the mediator was significantly different from zero [35,36]. These analyses were conducted both with BMI as a continuous mediator and dichotomized as obese versus not obese. The results were nearly identical and to be consistent with the outcome variable used in the logistic regression analyses (obesity), only the models that included obesity as a dichotomized indicator are presented. Finally, given the small amount of missing data (less than 1% for most variables, see Table 1), no systematic procedures for handling missing data were used, and cases with missing data were simply excluded from the final multivariate models. All analyses were conducted using SPSS 17.0.

**Table 1:** Demographic characteristics, Body Mass Index, sitting time and physical activity by sex among a cohort of Mexican American adults over age 18 (N=11,268)

	**N**	**Total**	**men**	**women**	***pa***
Participants (%)	11,268		18.9	81.1	<.001
Age in Years (Mean, SD)	11,268	40.0 (13.5)	41.6 (14.7)	39.6 (13.2)	<.001
Proportion of life lived in the U.S. (%)	11,268	52.2	58.1	50.8	<.001
Currently Employed (%)	10,688	41.1	70.3	34.1	<.001
High school or less (%)	11,242	51.7	50.9	51.8	.438
*Health outcomes*					
BMI (Mean, SD)	10,822	30.25 (6.40)	29.41 (5.48)	30.21 (6.56)	<.001
Obese (%)	10,822	45.5	38.9	45.9	<.001
Overweight, not obese (%)	10,822	35.3	42.6	33.1	<.001
Diabetes (%)	11,268	12.1	13.0	11.9	.144
Hypertension (%)	11,268	16.4	16.4	16.6	.875
Hypercholesterolemia (%)	11,268	16.2	18.4	15.7	.002
*Family History*					
Diabetes (%)	11,268	38.5	34.3	39.5	<.001
Hypertension (%)	11,268	30.9	21.9	33.0	<.001
Hypercholesterolemia (%)	11,268	12.8	7.6	14.0	<.001
Sitting hrs/day (Mean, SD)	11,256	3.69 (2.39)	3.90 (2.69)	3.64 (2.32)	<.001
Sitting mins/day (Mean, SD)	11,256	221.4 (143.5)	234.0 (161.4)	218.4 (138.9)	<.001
Strenuous PA min/day (Mean, SD)	11,126	7.2 (21.6)	21.0 (35.4)	3.70 (15.0)	<.001
Moderate PA min/day (Mean, SD)	11,090	10.2 (22.8)	15.5 (29.4)	8.8 (21.0)	<.001
Light PA min/day (Mean, SD)	11,197	50.4 (35.4)	29.5 (35.4)	55.2 (33.9)	<.001

^a^*p*-value based on either independent samples’ t-tests for continuous variables or chi-square tests for proportions.

## Results

### Participant characteristics

The mean age of participants was 40 years (SD=13.5), 81.1% were women and 24.7% were born in the U.S (see Table 1). A total of 41.1% of the participants were employed at the time of the interview (70.3% of males, 34.1% of females). Almost half (45.5%) of the participants had a BMI ≥ 30 kg/m^2^ and another 35.3% had a BMI between 25 and 30 kg/m^2^. A total of 12% of participants reported having ever been diagnosed as having diabetes. Approximately 16% of participants reported having ever been diagnosed with hypertension and high cholesterol. On average, participants reported sitting approximately 3.7 hours per day. Male participants reported sitting more hours per day, but also engaged in more moderate and strenuous physical activity compared to females. Men were less likely to have a BMI ≥ 30kg/m^2^, but more likely to report having high cholesterol (see Tables 1 and 2). Time spent sitting comprised, on average, 28.8% (SD=12.0%) of time spent sitting, sleeping and time spent engaging in physical activity combined.

**Table 2:** Demographic characteristics, Body Mass Index and physical activity by sitting time among a cohort of Mexican American adults (N=11,268)

	**1-2 hours**	**2-4 hours**	**more than 4 hours**	***p*-value**^**a**^
Proportion of participants (%)	27.0	41.1	32.0	
*Demographics*				
Female (%)	82.4	80.6	80.6	.056
Age in Years (Mean, SD)	40.9 (12.3)	40.0 (13.3)	39.1 (14.8)	<.001
Proportion of life lived in the U.S.	45.9	48.0	62.1	<.001
Currently employed (%)	43.1	40.0	41.3	.136
High school or less (%)	53.9	51.4	50.4	.005
Hours of Sleep/day (Mean, SD)	7.20 (1.39)	7.32 (1.36)	7.38 (1.53)	<.001
*Health outcomes*				
BMI (Mean, SD)	29.4 (5.6)	30.0 (6.1)	31.1 (6.9)	<.001
Obesity (%)	39.8	44.1	51.8	<.001
Overweight, not obese (%)	40.2	36.3	29.8	<.001
Diabetes (%)	10.8	11.5	13.8	<.001
Hypertension (%)	15.5	16.2	17.4	.030
Hypercholesterolemia (%)	15.6	16.4	16.4	.383
*Family History*				
Diabetes (%)	36.2	38.7	39.8	.002
Hypertension (%)	28.2	30.7	33.1	<.001
Hypercholesterolemia (%)	11.0	12.9	14.0	<.001
*Physical activity*				
Strenuous PA min/day (Mean, SD)	6.6 (20.9)	7.8 (23.2)	6.2 (20.0)	.474
Moderate PA min/day (Mean, SD)	11.0 (24.1)	9.8 (22.9)	9.5 (21.7)	.007
Light PA min/day (Mean, SD)	49.8 (35.7)	53.1 (35.6)	47.4 (35.5)	.007

^a^*p*-value based on trend analysis for linear contrasts with one-way ANOVA.

### Sitting time and health outcomes

*Obesity.* Multivariate logistic regression analyses showed that sitting time was associated with obesity. Compared to participants sitting 1-2 hours a day, participants who reported sitting more than 4 hours per day had higher odds of being obese (OR=1.55, 95% CI; 1.39, 1.73, *p*<.001). Participants sitting 3-4 hours a day also had higher odds of being obese compared to participants sitting 1-2 hours per day (OR=1.21, CI; 1.10, 1.34, *p*<.001) (see Table 3). Participants sleeping more hours per night and engaging in more moderate and strenuous physical activity had significantly lower odds of being obese. Older participants, females and participants who had lived a larger portion of their life in the U.S. had significantly higher odds of being obese.

**Table 3:** **Logistic regression predicting obesity, diabetes and hypertension from sitting time and other covariates among a cohort of Mexican origin adults (Final model N=10,688)**^**a**^

	**Obesity**^**b**^**OR (95% CI)**		**Diabetes OR (95% CI)**		**Hypertension OR (95% CI)**		**High cholesterol OR (95% CI)**	
Constant	0.34**		0.01**		0.00**		.011**	
*Demographics*								(1.06, 1.07)
Age	1.01**	(1.01, 1.01)	1.07**	(1.06, 1.07)	1.08**	(1.08, 1.09)	1.07**	
Female	1.18**	(1.06, 1.33)	0.94	(0.79, 1.12)	1.12	(0.95, 1.32)	0.90	(0.77, 1.05)
Educational attainment	0.96	(0.88, 1.04)	1.01	(0.89, 1.16)	1.05	(0.93, 1.18)	0.98	(0.87, 1.09)
Proportion of life in U.S.	1.89**	(1.66, 2.15)	1.11*	(1.02, 1.21)	1.18**	(1.05, 1.33)	1.02	(0.93, 1.13)
Currently Employed	0.99	(0.90, 1.07)	0.80**	(0.70, 0.93)	0.85*	(0.75, 0.97)	1.11	(0.99, 1.26)
Hours of sleep	0.94**	(0.91, 0.97)	0.97	(0.92, 1.02)	0.96	(0.92, 1.01)	0.99	(0.94, 1.03)
Family history (yes vs no)		N/A	2.59**	(2.28, 2.96)	1.70**	(1.50, 1.92)	1.95**	(1.67, 2.28)
*Physical Activity*								(0.73, 1.06)
Strenuous hrs/day PA	0.82**	(0.73, 0.93)	0.70**	(0.55, 0.89)	0.91	(0.75, 1.11)	0.88	
Moderate hrs/ day PA	0.83**	(0.74, 0.92)	0.99	(0.83, 1.18)	0.85*	(0.72, 0.98)	1.18*	(1.03, 1.37)
Light hrs/ day PA	1.03	(0.96, 1.11)	0.90	(0.81, 1.01)	1.01	(0.90, 1.10)	1.04	(0.94, 1.14)
*Sitting time*								
1-2 hours sitting/day	1.00		1.00		1.00		1.00	
2-4 hours sitting/day	1.21**	(1.10, 1.34)	1.04	(0.89, 1.23)	1.06	(0.92, 1.23)	1.07	(0.93, 1.23)
> 4 hours sitting/day	1.55**	(1.39, 1.73)	1.29**	(1.01, 1.52)	1.17*	(1.01, 1.37)	1.07	(0.93, 1.24)

*= coefficient significant on p≤.05, **=coefficient significant on p≤.01. **a** Models are multivariate logistic regression analyses controlling for participants age, sex, educational attainment, proportion of life lived in the U.S., employment status, hours of sleep, family disease history and strenuous, moderate and light physical activity. **b** Obesity was defined as having a Body Mass Index over 30 kg/m^2^ or not.

### Diabetes

Sitting time was associated with greater odds of having been diagnosed with diabetes. Participants sitting more than four hours per day had significantly higher odds (OR=1.29, 95% CI; 1.09, 1.52) of diabetes compared to participants who reported sitting 1-2 hours a day (see Table 3). Strenuous physical activity and currently being employed were associated with lower odds of having diabetes. Older age was associated with higher odds, as was having lived a larger proportion of one’s life in the U.S. Having a family history of diabetes was strongly (OR=2.59, 95% CI; 2.28, 2.96, *p*<.001) associated with diabetes.

### Hypertension

Sitting time was associated with greater odds of having reported a diagnosis of hypertension. Participants who reported sitting more than four hours per day had significantly higher odds of having hypertension compared to participants sitting 1-2 hours per day (OR=1.17, 95% CI=1.01, 1.37, *p=*.041) (see Table 3). Again, older age, living a larger portion of one’s life in the U.S., and having a family history of hypertension were associated with increased odds of having hypertension. Engaging in more moderate physical activity and currently being employed were associated with lower odds of hypertension.

### High cholesterol

Sitting time was *not* associated with increased odds of having high cholesterol (see Table 3).

### Mediation analyses

**Figure 1:**
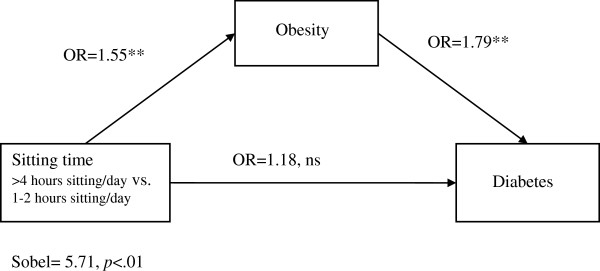
**Mediation of sitting time and diabetes through obesity among a cohort of Mexican Origin adults.** This figure shows the findings of a multivariate logistic regression analysis assessing if obesity mediated the association between sitting time and diabetes. The analyses controlled for participants’ age, sex, educational attainment, proportion of life lived in the U.S., employment status, family disease history and engagement in light, moderate and strenuous physical activity.

**Figure 2:**
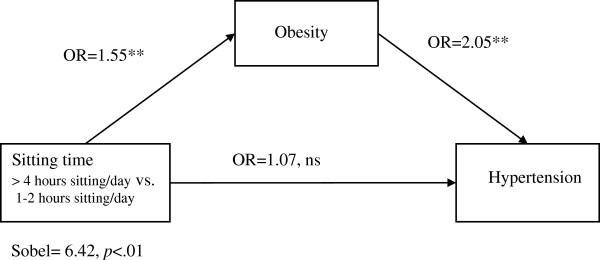
**Mediation of sitting time and hypertension through obesity among a cohort of Mexican Origin adults.** This figure shows the findings of a multivariate logistic regression analysis assessing if obesity mediated the association between sitting time and hypertension. The analyses controlled for participants’ age, sex, educational attainment, proportion of life lived in the U.S., employment status, family disease history and engagement in light, moderate and strenuous physical activity.

Because sitting time was not associated with high cholesterol, mediation analyses with obesity as a mediator were conducted only for models including hypertension and diabetes as outcomes. When including obesity as a predictor, the association of sitting time with diabetes and hypertension became non-significant (see Figures 1 and 2) for diabetes, OR=1.18 (95% CI: 0.99, 1.37, *p=*.068) and for hypertension OR=1.07 (95% CI= 0.92, 1.26, *p=*.376). A Sobel test [31,32] confirmed that obesity mediated the association between sitting time and diabetes (Sobel test= 5.71, *p*<.01) and between sitting time and hypertension (Sobel test= 6.41, *p*<.01).

### Exploratory subgroup analyses

To assess whether the high proportion of females in the study impacted the association between sitting time and health outcomes, a number of sex-based interactions were conducted. None of these interactions were significant, and there was no evidence that sitting time impacted women differently than men in the current study. Further, approximately 6.1% of participants were over the age of 65. Due to the increased loss of lean muscle mass at older age, effects of sitting time on health outcomes and the impact of BMI may differ among this subgroup. To assess this, analyses were conducted among the subgroup of participants 65 years and older. Among participants over the age of 65, sitting time was significantly associated with obesity (OR=1.66, 95% CI: 1.31, 2.10, *p<*.001), marginally associated with diabetes (OR=1.55, 95% CI: 0.97, 2.51, *p*=.068), but not significantly associated with hypertension, despite the similar odds ratio as the total study sample (OR=1.17, 95% CI: 0.75, 1.84, *p*=.432).

## Discussion

The aim of the current study was to assess whether daily hours of sitting time was an independent risk factor for increased chronic disease risk factors among a large cohort of Mexican origin adults. Daily hours of sitting were associated with increased odds of obesity, diabetes, and hypertension. Moreover, the effects of sitting time on diabetes and hypertension were mediated by obesity. These associations were maintained regardless of levels of light, moderate and strenuous physical activity, employment status, and other covariates.

To our knowledge, this is the first study to describe these associations among a large population of people of Mexican descent. A number of prior studies have assessed or hypothesized how time spent sitting may lead to adverse health outcomes, including biological as well as behavioral pathways (e.g. [38]). For example, prior research has suggested that leisure sitting time is associated with increased snacking, resulting in an increase in energy intake while energy output levels are decreased [39]. Although it is beyond the scope of the current study to assess these processes, several characteristics specific to this study’s participants may have impacted reported sitting time and the process in which sitting time can lead to adverse health consequences. Most participants were female, were not employed, had spent more than half of their lifetime outside of the U.S., were younger on average and had a higher BMI on average compared to participants in most prior studies (e.g. [8,24]). Further, it is important to note that the mean hours of daily sitting reported by the current study participants were substantially lower than those observed in prior studies that used objective measures of sitting time. For example, Johanssen and colleagues [40] found that even lean participants sat more than 10 hours per day on average. The mean hours sitting reported here more closely approximated self-reported leisure time sitting assessed in other studies [24], suggesting that the sitting time question used for this study may have assessed leisure time sitting rather than total time spent sitting.

Interestingly, we found that the effects of sitting time on diabetes and hypertension were mediated by obesity. Although some prior research has provided preliminary evidence that inclusion of indicators of obesity or adiposity attenuates the effect of sitting time on health outcomes [2,3,16], no mediation analyses had been reported to date. For example, Thorp and colleagues [8] reported that inclusion of an adiposity indicator (waist circumference) impacted the associations between sitting time and cardiovascular and metabolic outcomes. Among women in their study, the associations between sitting time and systolic blood pressure and HDL cholesterol disappeared, and the associations between sitting time and diastolic blood pressure and fasting plasma glucose were weakened. In addition, all associations between hours per day of TV viewing and health outcomes either weakened or disappeared with the inclusion of waist circumference [8].These findings and the findings of the current study are in contrast with reports that indicators of excess adiposity do not impact the association between sitting time and disease outcomes [21].

### Limitations

The current study has several limitations. First, the unique characteristics of the participants (i.e. a high proportion of females, immigrants, and the low employment rate) may limit the generalizability of the findings of the current study. Second, only BMI as an indicator of obesity was available, whereas waist circumference has been shown to be a stronger predictor of obesity- related health conditions [41]. Further, the survey is based on self-reported sitting time, although recent research has suggested that self-reported measures of sedentary behaviors may be appropriate for cross-sectional associations with health outcomes (as in the current study) [42]. In addition, the sitting time categories used in the current study are limited, as at the time of the study no uniform recommendations or biological mechanisms were known that could guide categorization of sitting time according to health consequences. Further, while the analyses controlled for employment status, the survey did not distinguish between occupational sitting and leisure time sitting. Also, due to the cross-sectional nature of the current study, our knowledge of temporality of exposure (time spent sitting) and disease occurrence is limited. For example, sitting time may precede disease outcomes, but may also be a consequence of disease onset and development. Finally, the survey items asked about *diagnosed* disease, and the percentages of undiagnosed disease were unknown in the current sample.

### Implications

The findings of this study provide relevant information for future programs and studies aimed at decreasing total hours and prolonged periods of sitting among Mexican origin populations. Intervention strategies can be as simple as standing up and briefly walking around while watching television or sitting at work for prolonged periods of time. Recently, Healy and colleagues [43] found that breaking up long periods of sitting have been associated with beneficial metabolic outcomes, independent of physical activity and total engagement in sedentary behavior.

## Conclusion

Although prior research has reported that indicators of excess adiposity did not attenuate the relation between sitting time and adverse health outcomes [21], the current study found that obesity mediated the associations of sitting time with diabetes and hypertension. Future research should evaluate in greater detail whether obesity mediates the association between sitting time and cardiovascular and metabolic disease outcomes, and whether interventions addressing sitting time are feasible and effective among Mexican origin populations.

## Competing interests

The authors declare that they have no competing interests.

## Authors’ contributions

HDdH developed the basic study concept in collaboration with co-authors, conducted the analyses and wrote the majority of the manuscript. AVW contributed to developing the study concept, edited all sections of the manuscript. LLS contributed to refining the study concept and contributed in writing all sections of the manuscript. MLB facilitated collaboration with the MD Anderson cancer center, provided feedback on the study concept and all sections of the manuscript. LMK conducted part of the analyses and edited all sections of the manuscript. All authors read and approved the final manuscript.

## Pre-publication history

The pre-publication history for this paper can be accessed here:

http://www.biomedcentral.com/1471-2458/12/896/prepub
